# Effects of low glycaemic index/low glycaemic load vs. high glycaemic index/ high glycaemic load diets on overweight/obesity and associated risk factors in children and adolescents: a systematic review and meta-analysis

**DOI:** 10.1186/s12937-015-0077-1

**Published:** 2015-08-25

**Authors:** Lukas Schwingshackl, Lisa Patricia Hobl, Georg Hoffmann

**Affiliations:** Department of Nutritional Sciences, Faculty of Life Sciences, University of Vienna, Althanstraße 14 (UZAII), A-1090 Vienna, Austria

## Abstract

The objective of the present systematic review and meta-analysis was to synthesize the available literature data investigating the effects of low glycaemic index/low glycamic load dietary regimens on anthropometric parameters, blood lipid profiles, and indicators of glucose metabolism in children and adolescents. Literature search was performed using the electronic databases MEDLINE, EMBASE, and the Cochrane Central Register of trials with restrictions to randomized controlled trials, but no limitations concerning language and publication date. Parameters taken into account were: body weight, body mass index, z-score of body mass index, fat mass, fat-free mass, height, waist cicrumference, hip circumference, waist-to-hip ratio, total cholesterol, LDL-cholesterol, HDL-cholesterol, triglycerides, diastolic and systolic blood pressure, fasting serum glucose, fasting serum insulin, HOMA-index, glycosylated haemoglobin, and C-reactive protein. Meta-analyses were performed for each parameter to assess pooled effect in terms of weighted mean differences between the post-intervention (or differences in means) of the low glycaemic index diets and the respective high glycaemic index counterparts. Data analysis was performed using the Review Manager 5.3. software. Nine studies enrolling 1.065 children or adolescents met the inclusion criteria. Compared to diets providing a high gylcaemic index, low glycaemic index protocols resulted in significantly more pronounced decreases in serum triglycerides [mean differences −15.14 mg/dl, 95 %-CI (−26.26, −4.00)] and HOMA-index [mean difference −0.70, 95 %-CI (−1.37, −0.04), fixed-effects model only]. Other parameters under investigation were not affected by either low or high glycaemic indices. The present systematic review and meta-analysis provides evidence of a beneficial effect of a low glycaemic index/load diet in children and adolescents being either overweight or obese. Regarding the limitations of this analysis, further studies adopting a homogenous design are necessary to assure the present findings. Since low glycaemic index/load regimens were not associated with a deterioration of the outcome parameters, these diets should not be categorically excluded when looking for alternatives to change lifestyle habits in this age group.

## Introduction

According to the World Health Organization (WHO), overweight and obesity remain the leading cause for premature death worldwide [1]. Especially alarming is the increasing number of children who are either overweight or obese. In the United States, the prevalence of childhood obesity nearly tripled between 1980 and 2000 [2]. Because of the fact that overweight children are about 30-50 % more likely to suffer from comorbidities such as metabolic syndrome in adulthood than those with normal weight, it is of utmost importance to treat overweight and obesity as soon as possible [3]. Weight management programs designed for the age group of children and adolescents often focus on a reduced fat and/or carbohydrate intake. Diets providing a low glycaemic index (LGI)/low glycaemic load (LGL) seemed to have promising effects on weight reduction, dyslipidaemia and blood glucose values.

The term „glycaemic index“ (GI) was introduced by Jenkins and co-workers in 1981 [4] referring to the area under the blood glucose curve measured two hours after consuming 50 g of test carbohydrates in relation to the results obtained by 50 g of glucose or white bread. In 1997 [5, 6] the term glycaemic load (GL) was introduced to quantify the overall glycaemic effect of food with respect to its specific carbohydrate content in typically consumed quantities (i.e. for a specific food, GL equals GI multiplied by the carbohydrate density of the food, usually given as g carbohydrate per 100 g serving). In a meta-analysis published in 2003, LGI diets exerted significant benefits on glycosylated haemoglobin (HbA1c) in type 1 and type 2 diabetics as compared to high GI (HGI) regimens [7]. These results could be confirmed by others demonstrating LGI diets being superior to their HGI counterparts with respect to HbA1c, serum fasting glucose (FG), body weight, fat mass, body mass index (BMI), total cholesterol (TC), LDL-cholesterol (LDL-C), and C-reactive protein (CRP) in overweight and obese study participants being otherwise healthy or diabetic [8–14]. Furthermore, a meta-analyses of cohort studies revealed that the highest category of GI/GL was associated with a significantly increased risk for the development of type 2 diabetes [15]. A correlation between GI/GL and risk of coronary heart disease was postulated as well [16].

Most of the epidemiologic data and intervention studies focus on the effects of LGI/LGL diets in adults. In the present systematic review, we investigated the impact of LGI and LGL protocols in randomized controlled trials on anthropometric parameters, blood lipid profiles, and indicators of glucose metabolism in children and adolescents with a mean age below 18 years.

## Methods

This systematic review is recorded in the PROSPERO International Prospective Register of Systematic Reviews (crd.york.ac.uk/prospero/index.asp) with the registration number CRD42015016799.

### Literature search

Queries of literature were performed using the electronic databases MEDLINE (between 1966 and February 2015), EMBASE (between 1980 and February 2015), and the Cochrane Central Register of trials (until February 2015) with restrictions to randomized controlled trials and age birth to 18 years, but no limitation to language and publication date. Search terms were: “glycaemic index” and “glycaemic load” as well as “glycemic index” and “glycemic load”. Selected articles were screened and sorted out if not all inclusion criteria were met. However, reference lists from retrieved articles were checked to search for further relevant studies, and systematic reviews and meta-analysis were searched as well. This systematic review was planned, conducted, and reported in adherence to standards of quality for reporting meta-analyses [17].

### Study selection

To be included in this systematic review, studies had to fulfil all of the following criteria: (1) randomized controlled trials (RCTs); (2) humans; (3) mean age of subjects < 18 years; (4) comparing a LGI or LGL with a HGI or high GL (HGL) dietary pattern; GI and/or GL values must have been reported; (5) report of post mean or mean of two time point values with standard deviation or basic data to calculate these parameters must have been given; (6) assessment of the “outcome of interest” markers: BMI, body weight, height, waist circumference (WC), hip circumference (HC), waist to hip ratio (WHR), fat mass, fat-free mass (FFM), TC, LDL-C, HDL-C, triglycerides (TG), diastolic blood pressure (DBP), systolic blood pressure (SBP), FG, fasting serum insulin (FI), HOMA-Index (HOMA), HbA1c, and CRP.

### Risk of bias assessment

**Fig. 1:**
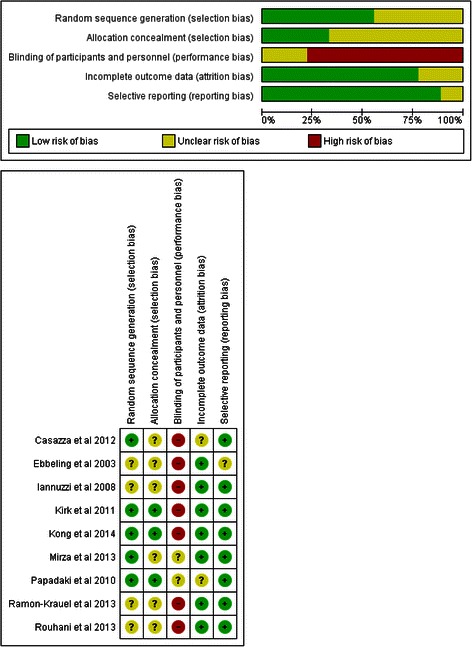
Risk of bias

Risk of bias was assessed by two authors (LS, GH) using the Risk of bias assessment tool provided by the Cochrane Collaboration. With this tool, the following sources of bias were detected: selection bias, performance/detection bias, attrition bias, reporting bias and other bias (such as contamination via mixing interventions and controls) [18, 19] (Fig. 1).

### Data extraction and statistical analysis

The following data were extracted from each study: the first author’s name and the year of publication, duration of the study, number, age and sex of participants, baseline BMI or BMI z-score, dietary descriptions with GI and GL values, amount of energy (kcal) and drop-out rate as well as outcomes and post mean values or differences in mean of two time point values with corresponding standard deviation. According to the Cochrane Collaboration, it is legit to use both the post-intervention values and differences in means in a meta-analysis [20]. For each outcome parameter a meta-analysis was created to compare the pooled weighted means at the endpoint of the studies or weighted mean differences from the LGI/LGL and HGI/HGL diet groups. All data were analyzed using the software REVIEW MANAGER 5.3. provided by the Cochrane Collaboration (http://tech.cochrane.org/revman). Forest plots were generated to illustrate the study-specific effect sizes along with a 95 %-CI. X^2^-tests were performed to examine the heterogeneity of the present data results. The I^2^ parameter was used to estimate the inconsistency of the results with I^2^ > 50 % was defined in advance to represent substantial heterogeneity.

Data extraction was conducted independently by all authors, with disagreements resolved by consensus.

### Specific data handling

In one of the studies included for meta-analyses [21], two different sets of LGI vs. HGI comparisons were designed distinguished by their protein content. Both designs were included in the study by comparing low protein/LGI with low protein/HGI and high protein/LGI with high protein/HGI separately.

## Ethical approval

Not required for this systematic review.

## Results

### Literature search and study characteristics

**Fig. 2:**
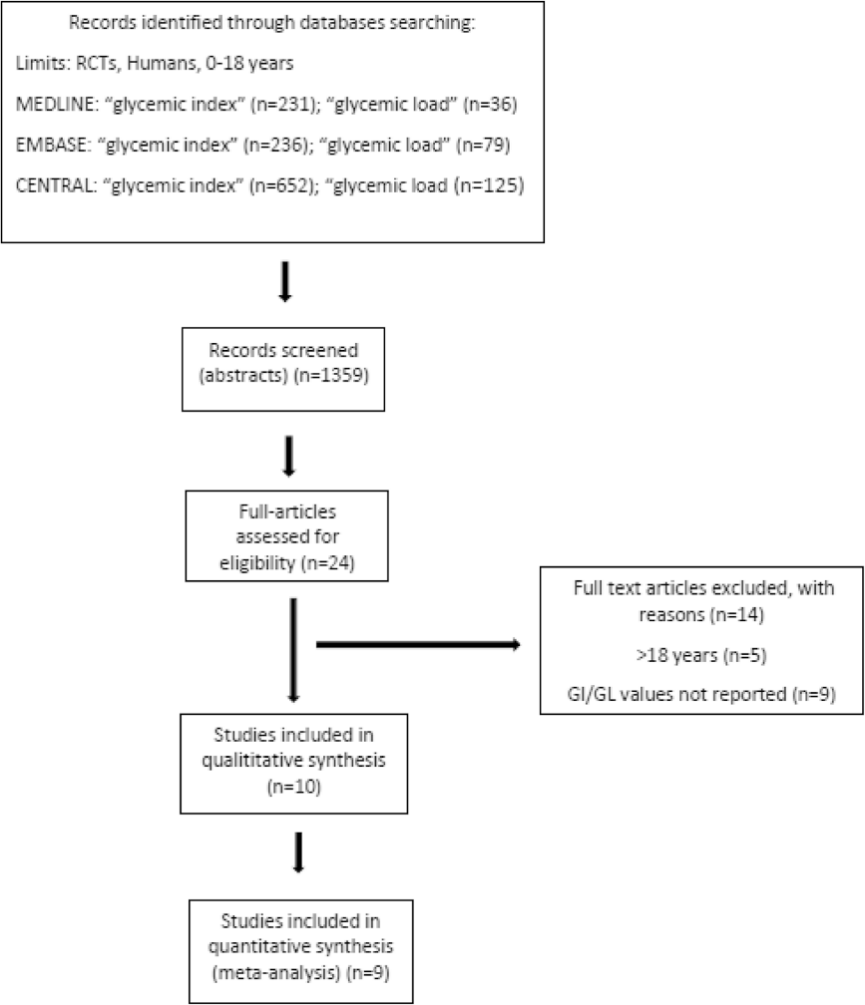
Flow diagram

A total of nine studies enrolling 1.065 subjects extracted from 1.359 articles met the inclusion criteria and were enclosed for meta-analyses [21–29]. The detailed steps of the systematic article search and selection process are given as a flow chart in Fig. 2.

All nine studies were randomized controlled trials (RCTs) with a duration between 10 and 96 weeks and a publication date between the years 2003 and 2014. General study characteristics are summarized Table 1. The data by Damsgaard et al. [30] were not suitable for meta-analyses, since not enough information was given by the authors to calculate standard deviations. For the other trials, the pooled estimates of effect size for the results of LGI/LGL compared to HGI/HGL for all outcome parameters are summarized in Table 2.

**Table 1:** General characteristics of randomized controlled intervention trials included in the meta-analysis

Reference	Participants	Age (yrs)	Duration (weeks)	Dietary intervention (as indicated by the investigators)	LGI, LGL	Energy amount (end of the study), or energy restriction	Drop Out
	Baseline BMI (kg/m^2^)	Female (%)			HGI, HGL		
		Male (%)					
	% Diabetics						
Casazza et al. 2012 [22]	26	12.4	16	Specialized diet	346, 129	2019 kcal	0 %
	BMI z-score: 2.4	100 %		Standard diet	503, 255 (bread reference)	2058 kcal	
	0 %	0 %					
Ebbeling et al. 2003 [23]	16	16.9	24	Reduced glycemic load diet vs	53, 68 (g/1000 kcal)	1621	12.5 %
	34.9	69 %				1439	12.5 %
	0 %	31 %		Low-fat diet	56, 77 (g/1000 kcal)		
Ianuzzi et al. 2008 [24]	26	Range: 7-13	24	diet high glycemic index vs	60	n.d.	0 %
	28.3	53.8 %			90		
	0 %	46.2 %		diet low glycemic index			
Kirk et al. 2012 [25]	66	9.8	48	Low carbohydrate diet vs	<55, 73.2 (g/1000 kcal)	1950 kcal	25.7 %
	29.5	63.6 %		Portion controlled diet		1900 kcal	9.6 %
	0 %	36.4 %			>70, 74.0 (g/1000 kcal)		
Kong et al. 2014 [26]	104	16.8	24	Low glycemic index diet vs	74.4, 117.7	1565 kcal	34.6 %
	30.9	56.7 %			76.8, 106.3	1981.6 kcal	48.1 %
	0 %	43.3 %		Control diet			
Mirza et al. 2013 [27]	113	11.65	96	Low glycemic load diet vs	55.5, 77.2	1148 kcal	52.6 %
	30.6	49 %		low fat diet	(g/1000 kcal)	1146 kcal	57 %
	0 %	51 %			54.4, 73.6 (g/1000 kcal)		
Papadaki et al. 2010 [21]	647	12.15	26	Low protein/LGI vs	60.8, 153.4	1692.4 kcal	37 %
	21.8	43.3 %		Low protein/HGI	62.9, 123.9	1382.6 kcal	48 %
	0 %	56.7 %		High protein/LGI vs	56.9, 105.0	1494.5 kcal	42 %
				High protein/HGI	63.9, 128.0	1643.9 kcal	39 %
Ramon-Krauel et al. 2013 [28]	17	12.8	24	LGI diet vs	54.6, 56.4	1271 kcal	12.5 %
	32.65	17.6 %		Low fat	(g/1000 kcal)	1422 kcal	0 %
	0 %	82.4 %			60.2, 70.4 (g/1000 kcal)		
Rouhani et al. 2013 [29]	50	13.89	10	LGI vs	43.22	1503 kcal	24 %
	n.d.	100 %		Healthy nutrition recommendations	46.70	1532 kcal	12 %
	0 %	0 %					

*BMI* = body mass index; *HGI* = high glycaemic index; *HGL* = high glycaemic load; *LGI* = low glycaemic index; *LGL* = low glycaemic load; *LP* = low protein

**Table 2:** Pooled estimates of effect size for the results of low glycaemic index/low glycaemic load compared to high glycaemic index/high glycaemic load

Outcome parameter (I^2^)	Mean difference	95 % confidence interval	*p*-Value	No. of studies	Sample size	I^2^ (%)
Body mass index, kg/m^2^	−0.54	(−1.19, 0.12)	0.11	7	836	13
Body mass index, kg/m^2^^1^	−1.00	(−2.31, .0.31)	0.13	5	119	24
Body mass index z-score	−0.09	(−0.19, 0.01)	0.08	4	777	0
Body mass index z-score ^1^	−0.11	(−0.23, 0.02)	0.10	2	40	0
Weight, kg	−0.10	(−1.86, 1.67)	0.92	5	794	9
Weight, kg ^1^	−0.37	(−2.82, 2.08)	0.77	3	103	0
Waist circumference, cm	−1.23	(−3.23, 0.77)	0.23	5	794	0
Waist circumference, cm ^1^	−1.40	(−5.46, 2.66)	0.50	3	103	0
Hip circumference, cm	−1.07	(−3.25, 1.12)	0.34	3	664	0
Hip circumference, cm ^1^	−8.00	(−23.5, 7.50)	0.31	1	16	NA
Wast-to-hip ratio	0.00	(−0.02, 0.01)	0.53	3	664	13
Wast-to-hip ratio ^1^	0.00	(−0.05, 0.05)	1.00	1	16	NA
Body fat, kg	−0.43	(−2.01, 1.14)	0.59	4	777	40
Body fat, kg ^1^	0.44	(−1.52, 2.40)	0.66	2	87	28
Fat mass, kg	−1.04	(−2.50, 0.43)	0.17	4	689	26
Fat mass, kg ^1^	−1.87	(−3.96, 0.22)	0.08	2	42	64
Fat-free mass, kg	0.81	(−1.10, 2.72)	0.40	2	647	71
Diastolic blood pressure, mm Hg	0.65	(−2.11, 3.41)	0.64	4	213	39
Systolic blood pressure, mm Hg	1.63	(−1.52, 4.78)	0.31	4	213	0
LDL-cholesterol, mg/dl	−1.61	(−8.09, 4.87)	0.63	4	237	0
HDL-cholesterol, mg/dl	0.27	(−2.13, 2.66)	0.83	5	263	0
Triglycerides, mg/dl	−15.14	(−26.26, −4.00)	0.008	5	263	0
Total cholesterol, mg/dl	−3.72	(−11.71, 4.27)	0.36	4	246	0
C-reactive protein, mg/l	0.58	(−0.25, 1.41)	0.17	1	26	NA
HOMA index	−0.70	(−1.37, −0.04)	0.04	4	172	30
Fasting serum insulin, μU/ml	2.67	(−6.32, 0.9)	0.15	3	109	0
Fasting serum glucose, mg/dl	−0.13	(−2.25, 1.98)	0.90	4	213	0

I^2^ = inconsistency (heterogeneity); *NA* = not applicable

1 For anthropometrical parameters, sensitivity analyses were performed including only trials investigating the effects of glycaemic index/load on children and adolescents being either overweight and/or obese (no such data were available for fat-free mass)

### Anthropometric parameters

None of the anthropometric data was affected significantly by an LGI/LGL dietary protocol (Table 2). Since most of the data came from the DIOGenes Study [21] enrolling many children with normal weight, sensitivity analyses were performed including only trials investigating the effects of LGI/LGL vs. HGI/HGL on children and adolescents being either overweight and/or obese. However, still no significant differences were found between the two dietary regimens (Table 2).

### Blood lipids

**Fig. 3:**
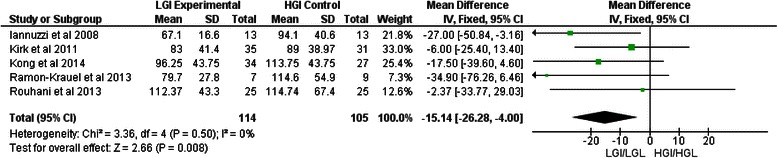
Forest plot showing pooled WMD with 95 % CI for triglycerides (mg/dl) for five randomized controlled LGI/LGL diets. For each LGI/LGL study, the shaded square represents the point estimate of the intervention effect. The horizontal line joins the lower and upper limits of the 95 % CI of these effects. The area of the shaded square reflects the relative weight of the study in the respective meta-analysis. The diamond at the bottom of the graph represents the pooled WMD with the 95 % CI for the six study groups. HGI = high glycaemic index; HGL = high glycaemic load; LGI = low glycaemic index; LGL = low glycaemic load; WMD = weighted mean difference

TG levels were significantly more decreased in the LGI/LGL groups as compared to their HGI/HGL counterparts [MD −15.14 mg/dl, 95 %-CI (−26.26, −4.00), *p* = 0.008] (Fig. 3). GI did not influence any of the other blood lipid values extracted for this systematic review (Table 2).

### Parameters of glucose control

**Fig. 4:**
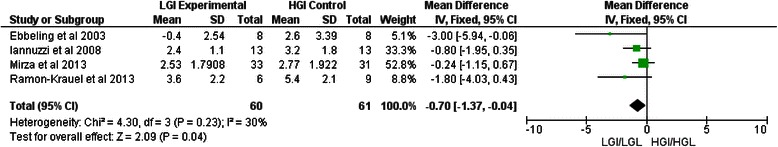
Forest plot showing pooled WMD with 95 % CI for HOMA-Index for four randomized controlled LGI/LGL diets. For each LGI/LGL study, the shaded square represents the point estimate of the intervention effect. The horizontal line joins the lower and upper limits of the 95 % CI of these effects. The area of the shaded square reflects the relative weight of the study in the respective meta-analysis. The diamond at the bottom of the graph represents the pooled WMD with the 95 % CI for the four study groups. HGI = high glycaemic index; HGL = high glycaemic load; LGI = low glycaemic index; LGL = low glycaemic load; WMD = weighted mean difference

Decreases in HOMA-index [MD −0.70, 95 %-CI (−1.37, −0.04), *p* = 0.04] were significantly more pronounced following an LGI/LGL diet as compared to the HGI/HGL protocols (Fig. 4) when fixed-effects models were applied. Following random-effects data synthesis, results were not statistically significant [MD −0.87, 95 %-CI (−1.75, 0.02), *p* = 0.06].

## Discussion

By synthesizing data of nine randomized controlled trials, the present meta-analysis provides evidence that a low GI/GL diet may exert beneficial effects on TG and HOMA-index in children and adolescents as compared to a high GI/GL dietary approach.

Overweight and obesity in children and adolescents represent a worldwide problem with grave consequences for those affected. Increased body fat mass is considered to be a major risk factor for the development of cardiovascular and metabolic diseases such as type 2 diabetes. Lifestyle modifications aiming at a normalisation of body weight with subsequent weight management are a core principle in primary and secondary prevention for those being either overweight or obese. The influence of variations in macronutrient compositions on body weight indices is discussed controversially both in adults as well as in children and adolescents. A number of dietary regimens differing in macronutrient content were compared in a systematic review by Gow and co-workers [31] with respect to their effects on children and adolescents with overweight and obesity. Results suggest that expressive indicators such as BMI z-score will show positive developments regardless of the dietary macronutrient composition as long as a hypocaloric diet is given. According to Gow et al., short-term advantages of a low carbohydrate regime as compared to high carbohydrate counterparts were no longer detectable in long-term follow-up investigations. In interventions by Demol et al. [32] and by Krebs et al. [33], BMI z-score was found to be decreased by an average of 0.25 following a low carbohydrate regimen. In our own study, we could observe a tendency towards a more prominent reduction in BMI z-score of ~0.09 following a low GI/GL diet as compared to a high GI/GL protocol. Although these changes are smaller in scope and non-significant, the trials enrolled in the present meta-analysis are characterized by a longer running time of 24–96 weeks as compared to the studies by Demol et al. (12 weeks) and Krebs et al. (13 weeks), respectively. This finding can be interpreted as an indication that a special focus on GI/GL might exert long-term benefits regarding body weight management. However, none of the other anthropometric parameters observed in this meta-analysis was affected in a different manner by either GI/GL dietary regimens.

An increased level of TG is regarded to be a predictor of atherosclerosis and subsequent cardiovascular diseases. When compared to their normal weight peers, children with overweight (+0.21 mmol/L) or obesity (+0.26 mmol/L) were reported to have significantly increased serum TG concentrations [34]. In the present meta-analysis, reduction in TG levels was significantly stronger following low GI/GL diets with improved values averaging 17 mg/dL (~0.17 mmol/L) which indicates a favourable change regarding the predicitve power of increased TG concentrations.

Another target of low GI/GL diets is insulin resistance, which is commonly associated with overweight and obesity and is regarded to be a precursor of the corresponding cardiovascular and metabolic consequences [35, 36]. Insulin resistance can be assessed via euglycaemic hyperinsulinaemic patch clamp, however, this procedure is not applicable for every trial due to its costs and invasiveness. Thus, it is more appropriate to use a surrogate marker when recording insulin resistance. Both surrogate markers used in our meta-analyses (HOMA index and fasting insulin) were classified to be inadequate in the assessment of insulin resistance in children and adolescents during a consensus conference on the topic in 2010 [37]. HOMA index is known to correlate well with FI [38], however, FI itself is regarded to be no suitable alternative for the gold standard [39–41]. Still, the fact remains that both parameters are measured in numerous intervention studies to assess the impact of lifestyle on insulin resistance. We could observe a reduction in HOMA-index that was significantly stronger (mean difference −0.70 units) in individuals subjected to a low GI/GL protocol as compared to its high GI/GL counterparts. Although there is no unanimously accepted cut-off value for HOMA-index to indicate insulin resistance in children and adolescents, some studies have suggested that values near to 3.0 might be adequate in the pediatric population [42]. Thus, changes like the ones found in the present study may be of clinical relevance. Decrease in FI was more pronounced, albeit not statistically significant, following a low GI/GL dietary protocol (−2.67 μU/mL) in the present analysis based upon three trials. Significantly improved FI values in a pediatric population following lifestyle interventions based upon changes in dietary habits and/or physical activity were described by Ho et al. [43, 44].

CRP is generally accepted as a biomarker for chronic low-grade inflammation usually observed in association with obesity, diabetes, or cardiovascular diseases [45, 46]. Observational as well as interventional studies reported a beneficial effect of a diet focusing on low GI/GL on serum CRP concentrations [12, 47]. This is in contrast to the results of Iannuzzi et al. [24] in the present analysis who could not observe a superior effect of low GI/GL on CRP when compared to high GI/GL diets. This seems to be more in line with data by Griffith et al. [48], who could not find a correlation between GI/GL and CRP in normal weight individuals with even lower levels of serum CRP in overweigth and obese individuals following a high GI/GL diet.

One major limitation of the present systematic review is the fact that there is only a low a number of studies assessing the specific outcome parameter chosen for data synthesis. This might explain that statistically significant differences between LGI/LGL and HGI/HGL could only be observed for TG and HOMA index, while other parameters were characterized by tendencies towards an improvement following diets adopting a low glycaemic index. In addition, there was a broad range of dietary measures with different setups for low or high GI/GL (e.g. via an additional variation in protein content) or the respective thresholds to distinguish between both protocols. Assessment of adhesion to dietary protocols, drop outs, and randomization of study participants were presented and grouped in different ways as well. This might be of special importance concerning a potential study immanent low adherence of volunteers to the prescripted dietary intervention. Although most of the studies enrolled in the present meta-analysis were characterized by a clear difference between initial LGI and HGI protocols, there was a tendency towards an approximation of dietary GI between groups at the end of the studies (e.g. Damsgaard et al. [30], who reported a dietary GI that was only four points lower in their LGI groups as compared to the HGI counterparts by the end of their trial). This can be explained by numerous reasons such as lack of taste stimuli affecting flavour of the diet. Although these limitations are characteristic for nutritional intervention studies [49], they nevertheless affect the validity of the data.

## Conclusion

The present systematic review and meta-analyses provide evidence of a beneficial effect of a low GI/GL diet in children and adolescents being either overweight or obese. Although no improvements could be found with respect to anthropometrical parameters such as body weight, BMI, BMI z-score, or waist circumference, a statistically significant positive impact was found for TG and HOMA index. Regarding the limitations of this analysis, the results are not solid enough to justify a clear recommendation. Further - esp. long-term - studies are necessary to assure the present findings. Since low GI/GL regimens were not associated with a deterioration of the outcome parameters, these diets should not be categorically excluded when looking for alternatives to change lifestyle habits in this age group.

## Abbreviations

BMI: Body mass index
CRP: C-reactive protein
DBP: Diastolic blood pressure
FFM: Fat-free mass
FG: Fasting glucose
FI: Fastin insulin
HbA1c: Glycosylated haemoglobin
HC: Hip circumference
HDL-C: High density lipoprotein-cholesterol
(H)GI: (high) glycaemic index
(H)GL: (high) glycaemic load
I^2^: Inconsistency
LDL-C: Low density lipoprotein-cholesterol
(L)GI: (low) glycaemic index
(L)GL: (low) glycaemic load
TC: Total cholesterol
SBP: Systolic blood pressure
TG: Triglycerides
WC: Waist circumference
WHR: Waist-to-hip ratio
(W)MD: (weighted) mean difference
